# Short and long-term outcomes after off-pump coronary endarterectomy stratified by different target vessels

**DOI:** 10.1186/s13019-022-02089-x

**Published:** 2022-12-25

**Authors:** Ying Fang, Hua Wei, Zhen Wu, Wei Song, Changcheng Liu, Haiyang Li, Chengxiong Gu

**Affiliations:** Department of Cardiac Surgery, Beijing Anzhen Hospital, Capital Medical University, Street No.2 Chaoyang District, Beijing, 100029 China

**Keywords:** Diffuse coronary artery disease, Coronary endarterectomy, Off-pump coronary artery grafting bypass, Clinical outcomes, Risk factors

## Abstract

**Background:**

The efficacy of off-pump coronary endarterectomy (CE) has been proven in patients with diffuse coronary artery disease (DCAD). However, the clinical benefits of of-pump CE stratified by different target vessels remain controversial. This retrospective study assessed the effect of the territory and number of CE on short- and long-term outcomes of DCAD.

**Methods:**

From January 2012 to December 2014, 246 patients undergoing off-pump coronary artery bypass grafting (OPCABG) + CE were included. The patients were grouped by the territory and number of CE. The primary endpoints were postoperative acute myocardial infarction (PMI) and long-term major adverse cardiovascular and cerebrovascular events (MACCE).

**Results:**

Sixty-five patients (26.42%) were in the left anterior descending branch (LAD) group (CE on LAD), 134(54.47%) in the right coronary artery (RCA) group (CE on RCA), and 47(19.10%) in the multi-vessels group. PMI in the LAD group, RCA group, and multi-vessels group were 3.08%, 6.72%, and 14.89%, respectively (*P* = 0.08). Multi-vessels CE (OR = 9.042, 95%CI 2.198–37.193, *P* = 0.002), CE-plaque length ≥ 3 cm (OR = 6.247, 95%CI 2.162–18.052, *P* < 0.001), and type 2 diabetes mellitus (2DM) (OR = 4.072, 95%CI 1.598–10.374, *P* = 0.003) were independent risk factors of PMI. The long-term (mean 76 months) MACCE in the LAD group, RCA group, and multi-vessels group were 13.85%, 17.91%, and 10.64%, respectively (*P* = 0.552). Cox analysis indicated that PMI (HR = 7.113, 95%CI 3.129–16.171, *P* < 0.001) and Age ≥ 65 years (HR = 2.488, 95%CI 1.214–5.099, *P* = 0.013) increased the risk of long-term MACCE.

**Conclusions:**

Multi-vessel CE and CE-plaque length ≥ 3 cm significantly increased risk of PMI after OPCABG + CE, but the territory and number of CE did not affect long-term MACCE.

## Background

Coronary endarterectomy (CE) has been a proven surgical technique for complete myocardial revascularization in diffuse coronary artery disease (DCAD) [1, 2]. Although increasing advances in surgical techniques and aggressive antithrombotic therapy, many cardiac surgeons avoided performing CE during Coronary artery bypass grafting (CABG) due to life-threatening postoperative myocardial infarction (PMI). In recent years, patients needed to CABG are often accompanied by more complex coronary artery lesions with multi-comorbidities because of increasing improvement in the percutaneous coronary intervention [3, 4]. So, CE is still a necessary surgical technique in selected cases. Many studies have focused on the safety and efficacy of CE with different techniques (off-pump vs on-pump, open-CE vs closed-CE) [5, 6]. However, the clinical benefits of CE stratified by territory and number of target vessels remain controversial. In this retrospective study, we investigated the effect of the territory and number of CE on short- and long-term outcomes in patients undergoing off-pump CABG + closed CE.

## Methods

### Patients and grouping

Between January 2012 and December 2014, 271 patients with DCAD who received off-pump CABG + CE were included in this study. Exclusion criteria included (1) Presence of one- or double-vessel disease; (2)SYNTAX score ≤ 23; (3) Urgent or emergent CABG; (4) Being concomitant valvular or aortic surgery; (5) Urgent switching from off-pump to on-pump CABG.

Referring to the exclusion criteria, a total of 246 patients were included in this retrospective study. According to the territory and number of CE, patients were divided into LAD group (CE on left anterior descending artery *n* = 65), RCA group (CE on the right coronary artery, *n* = 134), and Multi-vessels group [at least CE on two of LAD, RCA and left circumflex artery (LCX), *n* = 47].

The study was carried out following the Ethical Guideline of the Committee on Human Experimentation of our institution, and informed consent was obtained from the patients about the experimentation (Ethics approval number 2021050X).

### Surgical procedure

Off-pump CABG + CE was performed with a standard procedural protocol involving general anesthesia, median sternotomy, systemic heparinization with activated clotting time (ACT) > 300 s, and harvesting of the left internal mammary artery (LIMA) and saphenous vein (SV). LIMA was always anastomosed to LAD. The sequential SV graft was performed by proximal anastomosis to the ascending aorta firstly, and then distal anastomosis to LCX and RCA in succession. For selected patients with indications, the bilateral internal mammary arteries were used with a composite “Y” graft (LIMA in situ and RIMA grafted to LIMA) [7].

The CE was performed by as follow indications: (1) Coronary angiography indicating diffuse lesions with length > 2 cm, luminal diameter < 1 mm in the main coronary artery; (2) intraoperative inspection finding no suitable anastomotic location in the middle-distal of the coronary artery with a wide blood supply territory. The atherosclerotic plaque was removed using the closed-CE technique. The closed-CE was performed with the following steps: (1) Coronary knife to make a 5–8 mm arteriotomy at the anastomotic site; (2) Potts scissors to peel intimal plaque from adventitia on both sides of incision; (3) Pull plaque using forceps with the help of the reaction force form heart contracts. The distal end of the plaque needed to be completely peeled. The proximal end of plaque was peeled 3–5 mm and then cut off sharply to avoid competitive flow. A satisfactory standard of closed-CE was that the distal end of plaque was present of a translucent and rat-tail shape with blood back-flow via arteriotomy. If the distal end of plaque was not peeled completely, open-CE with extended arteriotomy and vein patch angioplasty was performed. The diameter of all target vessels for bypass grafting should be ≥ 1.5 mm referring to 1.5 mm coronary probe during operation. The postoperative antithrombotic strategy included (1) Unfractionated heparin to maintain ACT > 180 s from 3 h after surgery to extubation if bleeding < 300 ml in the first 3 h after surgery; (2) Low molecular heparin to bridge-therapy for 3 days after extubation; (3) Dual antiplatelet therapy at 6–24 h after surgery and continuing for one year; (4) One antiplatelet continuing for life.

### End-points

The primary end-points were PMI and long-term major adverse cardiovascular and cerebrovascular events (MACCE). MACCE included MI, death, redo revascularization, and stroke. The PMI was defined by (1) Postoperative cTnI more than 10 times the 99th percentile of the upper limit of normal reference value with normal preoperative cTnI value; (2) Ongoing evidence of myocardial ischemia including new pathology Q wave formation, coronary angiography confirming the presence of new coronary or grafts occlusion, imaging evidence of new viable myocardium loss or local wall motion abnormalities consistent with ischemic etiology [8].

The secondary end-points included death in hospital, duration of mechanical ventilation, and ICU stays, mechanical circulation support, and long-term survival. Follow-up was mainly performed by outpatient and telephone interviews, and ended in February 2021. All patients received guideline-directed medical therapy (GDMT) after surgery [9].

### Statistical analysis

Statistical analysis was performed using an extensively admissive software program SAS software (version 9.4; SAS Institute Inc., Cary, NC, USA). Data were presented as means ± standard deviation (SD) or median with interquartile range for continuous variables and as frequencies and percentages for categorical variables. The ANOVA test was used to address non-paired samples for the comparison of normally distributed parameters and the Wilcoxon rank-sum test for the comparison of non-parametric variables. The Chi-squared test and Fisher's exact test were applied for the comparison of categorical variables. The multivariate logistic regression analysis was performed to calculate odds ratio (OR) and 95% confidence interval (CI) for identifying the risk factors of PMI. Kaplan–Meier survival analysis was performed for long-term survival. Cox proportional hazards model was used for identifying risk factors of long-term MACCE. Differences were considered statistically significant only when the *p*-value was < 0.05.

## Results

### Baseline characteristics

A total of 246 patients with mean age (61.30 ± 8.47) years and 78.86% male were studied. Sixty-five patients (26.42%) were in the LAD group, 134 (54.47%) in the RCA group, and 47 (19.10%) in the multi-vessels group. The characteristic distribution of other demographics, cardiac function parameters, comorbidities in three groups was summarized in Table 1.

**Table 1:** Baseline characteristics in patients undergone off-pump CABG + CE

Variables	LAD group (*n* = 65)	RCA group (*n* = 134)	Multi-vessels CE group (*n* = 47)	*P* valve
Age (years)	60.40 ± 9.63	61.99 ± 7.92	60.57 ± 8.27	0.533
Gender (males; %)	53 (81.54)	106(79.10)	35 (74.47)	0.660
BMI (kg/m2)	26.31 ± 2.88	26.06 ± 2.69	26.27 ± 2.33	0.764
Smoking (%)	24 (36.92)	51 (38.06)	15 (31.91)	0.766
Hypertension (%)	39 (60.00)	71 (52.99)	34 (72.34)	0.067
Diabetes mellitus (%)	25 (38.46)	51 (38.06)	21 (44.68)	0.713
Prior MI (%)	2 (3.08)	9 (6.72)	3 (6.38)	0.657
Prior stroke (%)	14 (21.54)	18 (13.43)	7 (14.89)	0.351
PVD	26 (40.00)	40 (29.85)	20 (42.55)	0.182
LVEF	61.47 ± 7.76	61.81 ± 7.04	61.63 ± 7.50	0.953
LVEDD	49.87 ± 4.83	49.94 ± 4.90	49.50 ± 5.32	0.919
LDL-C	2.25 ± 0.63	2.62 ± 0.86	2.06 ± 0.58	0.171
HDL-C	0.95 ± 0.19	0.92 ± 0.18	0.94 ± 0.18	0.911
CCS class III or IV	12 (18.46)	39 (29.10)	8 (17.02)	0.134

*BMI* body mass index; *CCS class* Canadian Cardiovascular Classification for angina; *CE* coronary endarterectomy; *HDL-C* high density lipoprotein cholesterol; *LDL-C* low density lipoprotein cholesterol; *LVEDD* left ventricular end diastolic diameter; *LVEF* left ventricular ejection fraction; *MI* myocardial infarction; *PVD* peripheral artery disease

### Surgical characteristics and short-term outcomes

**Fig. 1:**
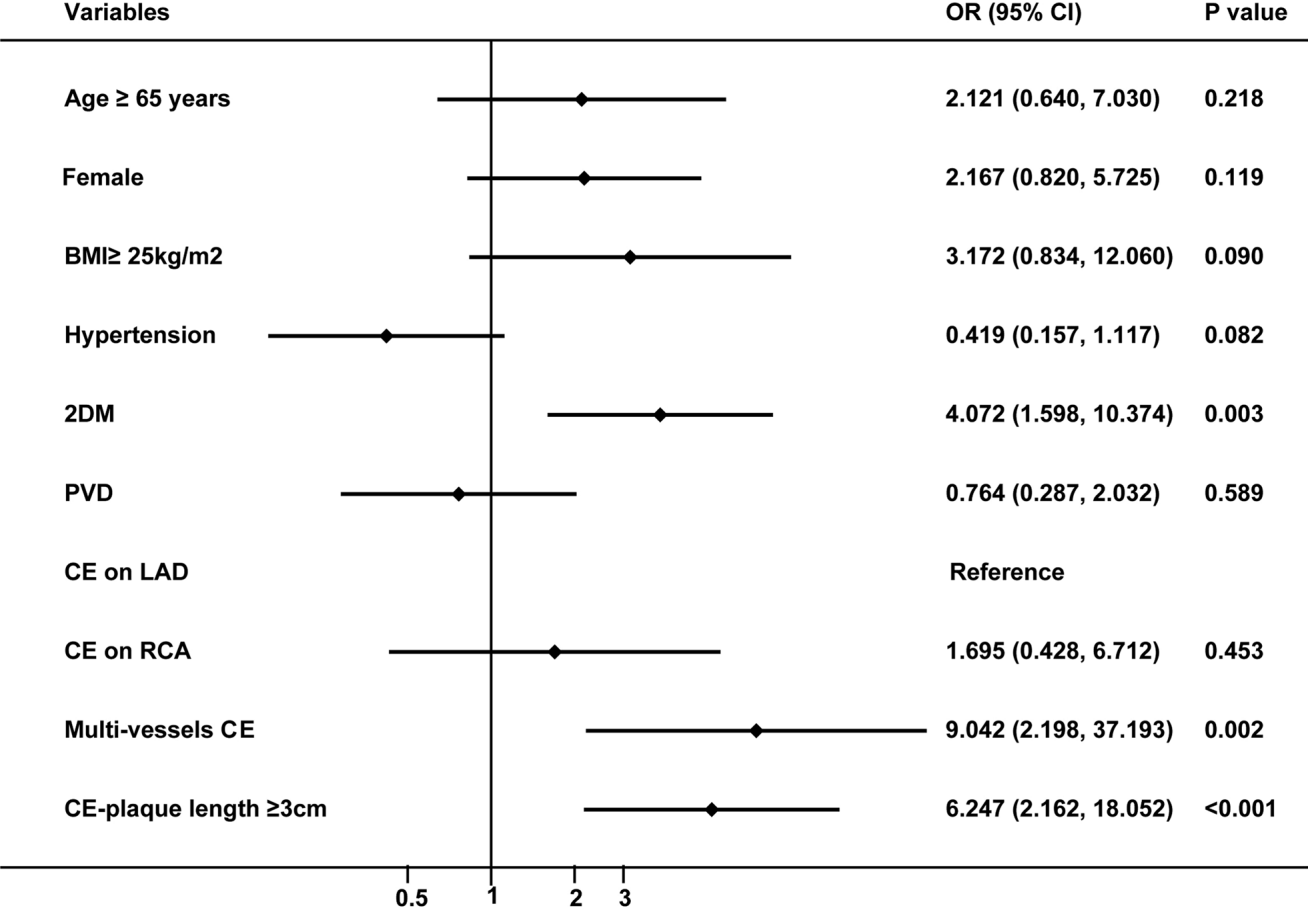
The multivariate logistic regression analysis for risk factors of PMI after off-pump CABG + CE. BMI, body mass index; CABG, coronary artery bypass grafting; CE, coronary endarterectomy; DM, diabetes mellitus; LAD, left anterior descending artery; PMI, postoperative myocardial infarction; RCA, right coronary artery

There was no difference in the number of graft anastomosis and LIMA grafting in the three groups. Mortality in hospital and PMI in all patients was 2.85% and 7.32%, respectively. Incidence of PMI in LAD group, RCA group, and multi-vessels group were 3.08%, 6.72%, and 14.89%, respectively, *P* = 0.08. The duration of mechanical ventilation, ICU stays, and mechanical circulation support was no different in the three groups. The details were shown in Table 2. The multivariate logistic regression analysis (Fig. 1) indicated that multi-vessels CE (OR = 9.042, 95%CI 2.198–37.193, *P* = 0.002), CE-plaque length ≥ 3 cm (OR = 6.247, 95%CI 2.162–18.052, *P* < 0.001), and 2DM (OR = 4.072, 95%CI 1.598–10.374, *P* = 0.003) were independent risk factors of PMI.

**Table 2:** Surgical characteristics and short-term outcomes after off-pump CABG + CE

Variables	LAD group (*n* = 65)	RCA group (*n* = 134)	Multi-vessels CE group (*n* = 47)	*P* valve
Number of graft anastomosis	3.91 ± 0.58	3.79 ± 0.52	3.89 ± 0.52	0.299
LIMA graft to LAD (%)	48 (73.85)	100 (74.63)	35 (74.47)	0.999
CE-plaque length ≥ 3 cm (%)	10 (15.38)	27 (20.15)	13 (27.66)	0.281
PMI	2 (3.08)	9 (6.72)	7 (14.89)	0.080
Death in hospital	0 (0)	4 (2.99)	3 (6.38)	0.096
Redo revascularization in hospital	0 (0)	1 (0.75)	1 (2.13)	0.415
Duration of mechanical ventilation (hours)	19 (17,22)	19 (15,23)	20 (15,22)	0.838
ICU LOS (hours)	24 (24,24)	24 (24,24)	24 (24,48)	0.553
Postoperative LOS (days)	7 (7,9)	7 (6,9)	8 (7,9)	0.132
*Mechanical circulation support*				
IABP	2 (3.08)	7 (5.22)	4 (8.51)	0.431
ECMO	0 (0)	1 (0.75)	1 (2.13)	0.415
Transfusion	8 (12.31)	22 (16.42)	9 (19.15)	0.218

*CE* coronary endarterectomy; *ECMO* extracorporeal membrane oxygenation; *IABP* intra-aortic balloon pump; *ICU* intensive care unit; *LAD* left anterior descending branch of coronary artery; *LIMA* left internal mammary artery; *PMI* postoperative myocardial infarction; *LOS* length of  stay

### Long-term outcomes

Follow-up was completed in 97% of patients. The median duration of follow-up was 79 (73, 86) months. The cumulative incidence of MACCE in the LAD group, RCA group, and multi-vessels group were 13.85%, 17.91%, and 10.64%, respectively (*P* = 0.522). The stroke rate in the LAD group was higher than in other groups (6.15% vs 0.75% vs 0%, *P* = 0.034). But repeat revascularization after angiography was no significant difference in the three groups. The long-term outcomes were summarized in Table 3.

**Table 3:** Long-term outcomes in patients undergone off-pump CABG + CE

Variables	LAD group (*n* = 65)	RCA group (*n* = 134)	Multi-vessels CE group (*n* = 47)	*P* valve
All-cause mortality	4 (6.15)	19 (14.18)	5 (10.64)	0.260
Myocardial infarction	0 (0)	2 (1.49)	0 (0)	0.999
Stroke	4 (6.15)	1 (0.75)	0 (0)	0.034
Repeat revascularization	1 (1.54)	3 (2.24)	0 (0)	0.817
MACCE	9 (13.85)	24 (17.91)	5 (10.64)	0.522

*MACCE* major adverse cardiovascular and cerebrovascular events

**Fig. 2:**
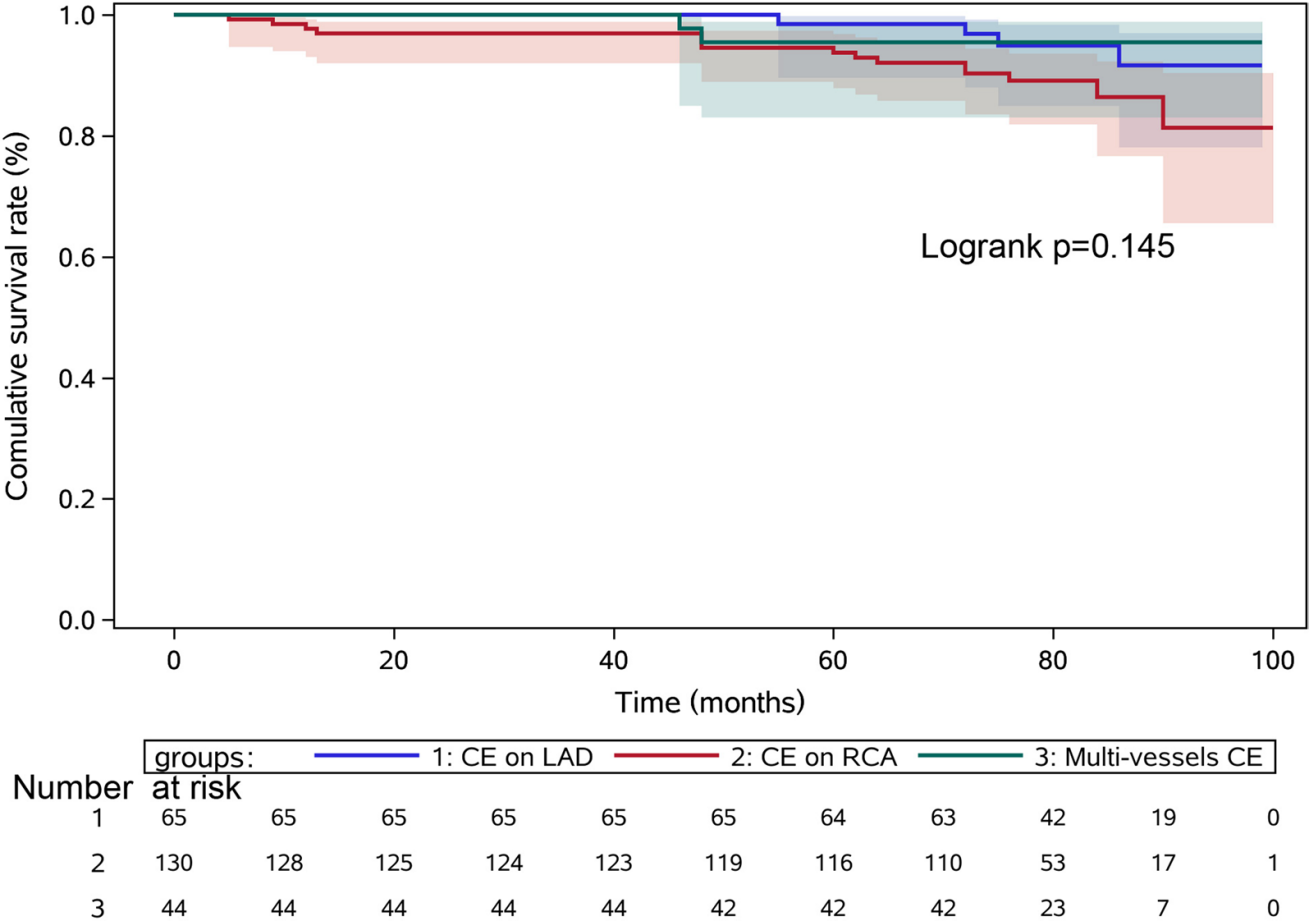
The Kaplan–Meier survival analysis for long-term survival rate after off-pump CABG + CE. CABG, coronary artery bypass grafting; CE, coronary endarterectomy

**Fig. 3:**
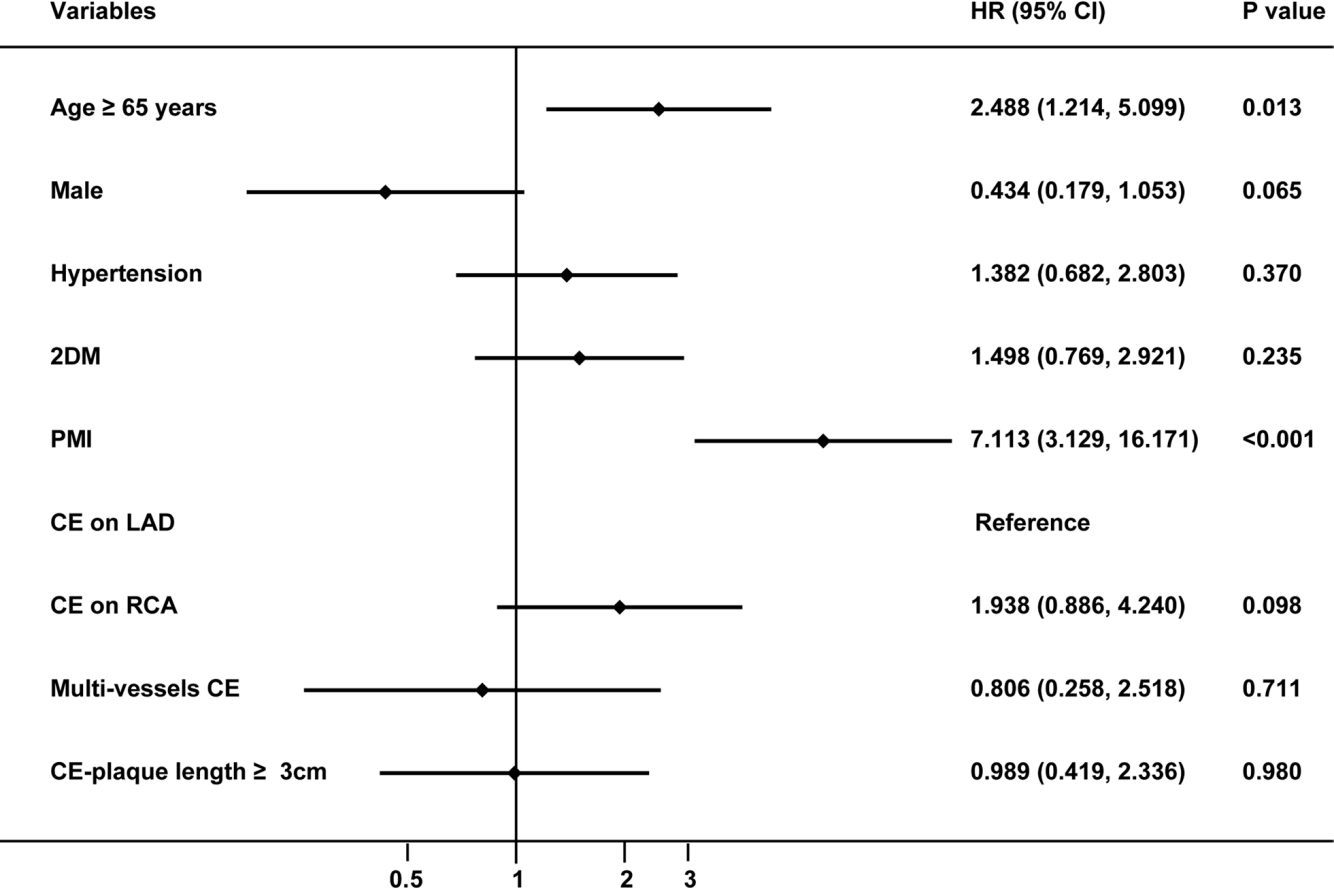
The Cox proportional hazards model for identifying risk factors of long-term MACCE after off-pump CABG + CE. CABG, coronary artery bypass grafting; CE, coronary endarterectomy; MACCE, major adverse cardiovascular and cerebrovascular events

Kaplan–Meier survival analysis (Fig. 2) indicated that the 8-year cumulative survival rate in the three groups was no different, log-rank *P* = 0.145. The COX regression analysis (Fig. 3) indicated that PMI (HR = 7.113, 95%CI 3.129–16.171, *P* < 0.001) and Age ≥ 65 years (HR = 2.488, 95%CI 1.214–5.099, *P* = 0.013) were independent risk factors of long-term MACCE.

## Discussion

This study investigated the effect of the number and territory of CE on clinical outcomes in patients undergoing off-pump CABG + CE. The main findings included that multi-vessels CE, CE-plaque length ≥ 3 cm, and 2DM were independent risk factors of PMI; long-term MACCE and survival were similar irrespective of the territory and number of CE.

In recent years, advancements in antithrombotic agents, surgical techniques, and perioperative care management maybe reduce the risk of morbidity and mortality in DCAD patients [10]. The incidence of PMI was 7.32% in this study, which was lower than that in a previous study [11]. In this study, we adopted a more radical antithrombotic strategy, namely dual antiplatelet + heparin in three days after surgery. But bleeding risk (requiring transfusion) was not significantly increased compared with previous studies [12].

Acute thrombosis is the most common cause resulting in PMI due to CE breaking the structural and functional integrity of coronary endothelium [13]. This study indicated that the more extensive CE (multivessel CE and CE-plaque length ≥ 3 cm) was performed, the more risk of PMI occurred, irrespective of the territory of CE. For patients undergone extensive CE, based on aggressive antithrombotic therapy, maintaining high blood perfusion of graft and coronary maybe reduce the risk of PMI after CE. In our previous study, prophylactic use of IABP during intraoperative remarkably reduced PMI in patients who suffered from extensive CE [14].

Furthermore, 2DM was also an independent risk factor of PMI. The main reason was that 2DM promoted a rapidly progress of coronary atherosclerosis [15]. Unfortunately, the prediabetes was also associated with a smaller coronary size and diffuse coronary narrowing [16]. So, the patients with coronary artery disease and 2DM have smaller-caliber vessels with more severe, extensive-diffuse atherosclerotic lesions. And previous studies indicated that the coronary artery caliber was the strongest predictors of perioperative mortality [17, 18]. Clinical management of DCAD complicated with 2DM should be strengthened for improving outcomes [19].

CE mainly affects early outcomes due to the high risk of thrombosis. Our results showed long-term outcomes were similar in terms of MACCE and survival in the three groups, although the incidence of stroke in the LAD group was significantly higher than the other two groups, which may be related to the high rate of preoperative history of stroke in the LAD group. So, regardless of the number and territory of CE, complete removing plaques at the distal of anastomosis site and complete revascularization were critical to maintain good run-off and improve long-term prognosis.

Mature CE technique is the guarantee of complete removing plaques. Surgical techniques treating DCAD include off-pump or on-pump open-CE and closed-CE. Previous studies indicated that the operative mortality was not statistically different between the on-pump CABG + CE versus off-pump CABG + CE groups or between open-CE versus closed-CE [5, 6]. In this study, the off-pump closed CE technique was used in the majority of patients. The key points of off-pump closed CE technique include (1) Mature atherosclerotic plaques, (2) Incision closing to distal segment of the plaque, and (3) gently pulling out plaque with help of counterforce from heart contraction.

Moreover, reendothelialization after CE also reduces the risk of long-term restenosis and thrombosis. Evidence from the animal model and human angiographic study has shown that neointima circumferentially covered the endarterectomies lumen [20, 21]. In a porcine coronary endothelial injury model, intimal regeneration has been observed at 35 days after coronary artery injury [22]. GDMT has also played an important role in improving long-term outcomes. Our results indicated that although 2DM was one of the risk factors causing PMI, it did not impact long-term MACCE.

## Limitations

Several limitations should be considered. First, the retrospective study was susceptible to inherent bias. Second, patients in the multi-vessels CE group were small. Multi-vessels CE was an independent risk factor causing PMI, but PMI was no difference between groups. Further study should enroll in more patients to testify these results. Third, coronary angiography was not routine follow-up content, coronary artery computed tomographic angiography will be needed to access graft patency in the different territory after CE.

## Conclusions

Whole removing coronary plaque in run-off and complete revascularization were critical to improving long-term outcomes in patients received off-pump CABG + closed CE, regardless of territory and number of CE. Multi-vessels CE and CE-plaque length ≥ 3 cm were independent risk factors causing PMI. The territory and number of CE did not affect long-term clinical outcomes.

## Data Availability

The datasets used are available from the corresponding author on reasonable request.

## Abbreviations

ACT: Activated clotting time
CABG: Coronary artery bypass grafting
CE: Coronary endarterectomy
DCAD: Diffuse coronary artery disease
DM: Diabetes mellitus
LAD: Left anterior descending artery
LCX: Left circumflex artery
LIMA: Left internal mammary artery
GDMT: Guideline-directed medical therapy
MACCE: Major adverse cardiovascular and cerebrovascular events
PMI: Postoperative acute myocardial infarction
RCA: Right coronary artery
SV: Saphenous vein
